# A Warning

**Published:** 1915-07

**Authors:** 

**Affiliations:** San Antonio


### A Warning.

It has been said that woman is the capital stock of the race, and that upon her rests its future integrity. Whatever else might be said about her, one single fact remains unchallenged through time, and that is, the crown of maternity is hers. Try to escape it if you will, the result will always be the same,—woman must bear the future generations. Unless reproduction is maintained at an equal or higher rate than the death rate, it is obvious that it would be but a question of time when the race would die out.

The question now arises, since the independence of woman is growing so rapidly, what is society doing to avert this impending disaster? Our economic and social conditions are driving women more and more into celibate lives, and married people, amongst the professional classes, into the adoption of family limitation. Verily the social calling of woman is becoming prostituted. What would be the result if all farmers ceased to produce crops and converted their acres to other purposes? Agriculture would die. Likewise, then, what is going to become of the race when all women voluntarily cease to become mothers?

To bring this matter home to you, we quote below some very pertinent statistics recently compiled by Prof. Robert J. Sprague from the Massachusetts vital statistics. This author states that, out of 200 children, 103 will be males and 97 females. Of the latter, 19 will die before maturity, leaving 78 for possible matrimony. Fifteen per cent of these will decline marriage, which reduces the number to become wives to 66. Twenty per cent of these will be sterile, which will make the number of actual child-bearing women 53. On these 53 women devolves the obligation of reproducing the original number of 200, or the rearing for each 3.7 children. These are plain facts, and cannot be overlooked. They make an eloquent appeal—"back to motherhood."

